# Fatal Errors

**Published:** 1881-03

**Authors:** Ad. Lippe

**Affiliations:** Philadelphia


### FATAL ERRORS.

BY AD. LIPPE, M.D., PHILADELPHIA.

It is a fatal error for a professedly homœopathic physician to administer crude doses of Chininum Sulfuricum for the cure of intermittent fever, or for any other purpose. Such practice is in absolute contravention of the strict inductive method of Hahnemann. Such practice is based on the fatal error that Quinine is a specific for intermittent fever; and this fatal error is the indisputable prerogative of the Common School of Medicine.

It was this very fatal error, as stated by Cullen in his "Materia Medica," that Peruvian Bark was a Specific for intermit tent fever which induced Hahnemann to clear up this fatal error. His investigations, consisting in the proving of Peruvian Bark on himself, fortunately for suffering humanity, showed clearly under what conditions Peruvian Bark became the curative remedy for that disease under the law of the similars. These first investigations of this great philosopher became the corner-stone of the strict inductive method of Hahnemann; on this corner-stone rests the magnificent structure of our healing art. The attempt to lead our healing art back into the dark ante-homœopathic teachings; the attempt to lay unholy hands on the corner-stone of our healing-art is tantamount to an attempt to destroy and set aside the strict inductive method of Hahnemann, and what then is left us? Nothing in fact, but the assumption of a name to which these men who attempt this progress backward have no claim whatsoever. Our literature is filled with incontrovertable evidence that the true healer can cure, always will cure, and always has cured, intermittent fever under strictly homœopathic treatment. There was the ever faithful Bœnninghausen who gave the profession his great work on intermittent fever, translated of late by Prof. Korndœrfer; there is our faithful Prof. H. C. Allen who has given us the well marked characteristic symptoms of the most important remedies, applicable under the law of the similars, for the cure of this disease; there are thousands of cases related in our journals showing the success of men who diligently selected their remedies for the cure of each individual case, under the homœopathic law and administered that remedy in appropriate doses, as taught us by the great master. With all these facilities offered to the busy practitioner, there is no sort of excuse left to the thoughtless pretenders when they commit the fatal error of leaving the system they profess to practice to fall into the fatal errors of the Common School of Medicine. Now for stubborn facts! Quinine, if homœopathically indicated, will cure any case of that disease when administered in a homœopathic dose; if not homœopathically indicated, the homœopathic dose does not cure. Nor do massive doses do more than semi-occasionally suppress the disease, leaving the sick, if even the attacks are stopped for a time, in a much worse conditlon than he was before; and in a really *deplorable* condition when these massive doses have been frequently and increasingly administered without even suppressing the attacks temporarily. We are induced to protest against this imposition practiced on unsuspecting persons who seek homœopathic treatment and are so cruelly deceived, while the brazen-faced impostor declares that he practices progressive homœopathy. Such victims not unfrequently return to a homœopathician who then finds them in a worse plight than he is accustomed to find patients who come to him from the allopathic school, uncured of the disease. It is humiliating to hear the reports of these victims; it is a deplorable sight to see these innocent suffering victims. And why is this thus? A worse than fatal error has been committed by public teachers in homœopathic colleges! When a professor charged with teaching homœopathy advises students to first suppress the intermittent fever attacks by administering the crude Quinine in doses of three grains and afterward to administer homœopathic remedies for the cure of the sick, he commits a gross and fatal error. Such practice is not only non-homœopathic, but the ill-advised young doctor will find it also utterly unsuccessful. He will then rush into further excesses, give larger doses and instead of becoming a healer and advancing our cause, he will cease to heal, and disgrace our school.

It is a fatal error for a Faculty to permit one of its members to teach a fatal error; and while some may teach homœopathy properly, they nevertheless innocently endorse both the teaching, and the teacher, of fatal errors.

It is a fatal error if the trustees of a homœopathic college allow such pernicious teachings. Homœopathic colleges have been chartered by a generous people through their Legislatures; and whether a charter has been designedly altered, whether the original charter called for the teaching of homœopathy *especially*, and was afterward amended so as merely to provide that homœopathy should "*also*" be taught, the fact remains the same, that homœopathy *should* be taught in that chartered institution. This is a peremptory command, and it is the solemn duty of the trustees, who represent the people under the charter by them granted, to see to it that this command is obeyed. There are men like Prof. Gonzalvo C. Smythe, A.M., M.D., the author of "Medical Heresies," lurking about, and exposing relentlessly the heresies observed in our school, seemingly in full earnest to declare homœopathy annihilated. Such men might show that a professor in a homœopathic college teaches, without any restraint or molestation, allopathic practice; that he has given up the strict inductive method of Hahnemann; that we therefore are no longer entitled to a school in which we were peremptorily commanded to teach homœopathy; and that we furthermore deserve to be mentioned only as a "Caricature in the history of medicine." Let us now, while it is yet time, correct this fatal error; let us denounce every homœopathic physician who administers Quinine for intermittent fever as an apostate; and a professor, who by his teaching leads the way to such abominable practice, as undeserving the trust he holds.

